# Elimination of HIV-1-infected cells by broadly neutralizing antibodies

**DOI:** 10.1038/ncomms10844

**Published:** 2016-03-03

**Authors:** Timothée Bruel, Florence Guivel-Benhassine, Sonia Amraoui, Marine Malbec, Léa Richard, Katia Bourdic, Daniel Aaron Donahue, Valérie Lorin, Nicoletta Casartelli, Nicolas Noël, Olivier Lambotte, Hugo Mouquet, Olivier Schwartz

**Affiliations:** 1 Virus and Immunity Unit, Department of Virology, Institut Pasteur, Paris 75015, France; 2 CNRS-URA 3015, Paris 75015, France; 3 Laboratory of Humoral Response to Pathogens, Department of Immunology, Institut Pasteur, Paris 75015, France; 4 CNRS-URA 1961, Paris 75015, France; 5 Université Paris Sud, UMR-1184, Le Kremlin Bicêtre 94276, France; 6 CEA, DSV/iMETI, Division of Immuno-Virology, IDMIT, Fontenay-aux-Roses 92260, France; 7 Inserm, U1184, Center for Immunology of Viral Infections and Autoimmune Diseases, Le Kremlin Bicêtre 94276, France; 8 APHP, Service de Médecine Interne–Immunologie Clinique, Hôpitaux Universitaires Paris Sud, Le Kremlin Bicêtre 94276, France; 9 Vaccine Research Institute, Creteil 94000, France

## Abstract

The Fc region of HIV-1 Env-specific broadly neutralizing antibodies (bNAbs) is required for suppressing viraemia, through mechanisms which remain poorly understood. Here, we identify bNAbs that exert antibody-dependent cellular cytotoxicity (ADCC) in cell culture and kill HIV-1-infected lymphocytes through natural killer (NK) engagement. These antibodies target the CD4-binding site, the glycans/V3 and V1/V2 loops on gp120, or the gp41 moiety. The landscape of Env epitope exposure at the surface and the sensitivity of infected cells to ADCC vary considerably between viral strains. Efficient ADCC requires sustained cell surface binding of bNAbs to Env, and combining bNAbs allows a potent killing activity. Furthermore, reactivated infected cells from HIV-positive individuals expose heterogeneous Env epitope patterns, with levels that are often but not always sufficient to trigger killing by bNAbs. Our study delineates the parameters controlling ADCC activity of bNAbs, and supports the use of the most potent antibodies to clear the viral reservoir.

## Abstract (web summary)

Broadly neutralizing antibodies (bNAbs) are promising as potential therapies targeting HIV-1 but their overall antiviral activity remains to be fully elucidated. Here the authors evaluate the ability of a panel of bNAbs to trigger antibody-dependent cellular cytotoxicity and identify the most effective antibody combinations.

## Graphical abstract

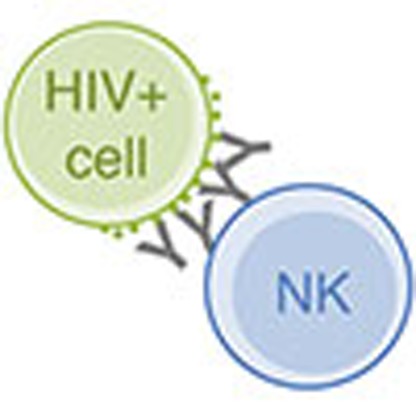

Two to four years post infection, rare HIV-1-positive patients develop a broadly serologic neutralizing activity against various viral strains^1^,^2^,^3^. The isolation and molecular characterization of bNAbs produced in these individuals have allowed the identification of five major ‘sites of vulnerability' on the HIV Env trimer^2^,^4^,^5^. Passive transfer of the most potent bNAbs provides both pre-exposure prophylaxis and treatment in macaque and humanized mouse models^3^,^4^,^5^. In HIV-1-infected individuals, a single infusion of the 3BNC117 bNAb, which targets the CD4-binding site on gp120, decreases viraemia for up to 28 days^6^. *In vivo*, the antiviral activity of bNAbs results from antigen-binding site-Env interactions that block entry of cell-free virions as well as viral cell–cell transmission^7^,^8^. Their activity is also highly dependent on the effector functions mediated by the Fc region, as demonstrated in animals using Fc-mutated bNAbs^9^,^10^,^11^.

Antibody effector functions include antibody-dependent cellular cytotoxicity (ADCC), mediated through binding of the Fc portion of antibodies to Fc receptors (FcRs) on effector cells including natural killer (NK) cells^12^,^13^,^14^. There is an increased interest in understanding the role of ADCC to prevent and control HIV-1 infection^13^,^14^. The presence of anti-Env IgG antibodies displaying ADCC in the absence of a strong IgA response is a main correlate of protection in the RV144 HIV-1 vaccine trial^15^,^16^. In HIV-infected individuals, the presence of ADCC antibodies often correlates with a slow disease progression^12^,^13^,^14^,^17^,^18^,^19^. An ADCC activity is also associated with reduced mortality in HIV-infected infants^20^. Serum ADCC-mediating antibodies target various Env epitopes including the variable loop 3 (V3), the constant region 1 (C1) and the CD4-induced (CD4i) region^21^,^22^ and likely exert significant immune pressure on the virus^21^. The ADCC activity of some anti-Env antibodies (including b12, 2G12, PGT126, as well as A32 that target a CD4i epitope) has been well studied^12^,^23^,^24^,^25^. These antibodies bind to Env glycoproteins at the cell surface and mediate their killing by NK cells. Interestingly, HIV-1 partly escapes ADCC. The HIV-1 Vpu and Nef proteins reduce the ability of some antibodies (targeting mostly CD4i epitopes) to perform ADCC^12^,^23^,^24^,^25^.

Cure strategies are aimed at targeting the latent HIV-1 reservoir within resting CD4^+^ T cells after viral reactivation^26^. bNAbs associated with viral inducers decrease rebound in humanized mice, through partly understood mechanisms that may include direct elimination of infected cells^27^. It is thus important to examine the competence of bNAbs to perform ADCC, to understand the underlying mechanisms and to determine whether ADCC-potent bNAbs may be used to purge or reduce the size of the latent reservoir. We identify here a subset of bNAbs that bind and kill HIV-1-infected cells through NK engagement. Furthermore, reactivated infected cells from HIV-positive individuals expose heterogeneous Env epitope patterns, with levels that are sufficient to trigger ADCC by bNAbs.

## Results

### Identification of bNAbs that kill HIV-1 infected lymphocytes

**Figure 1:**
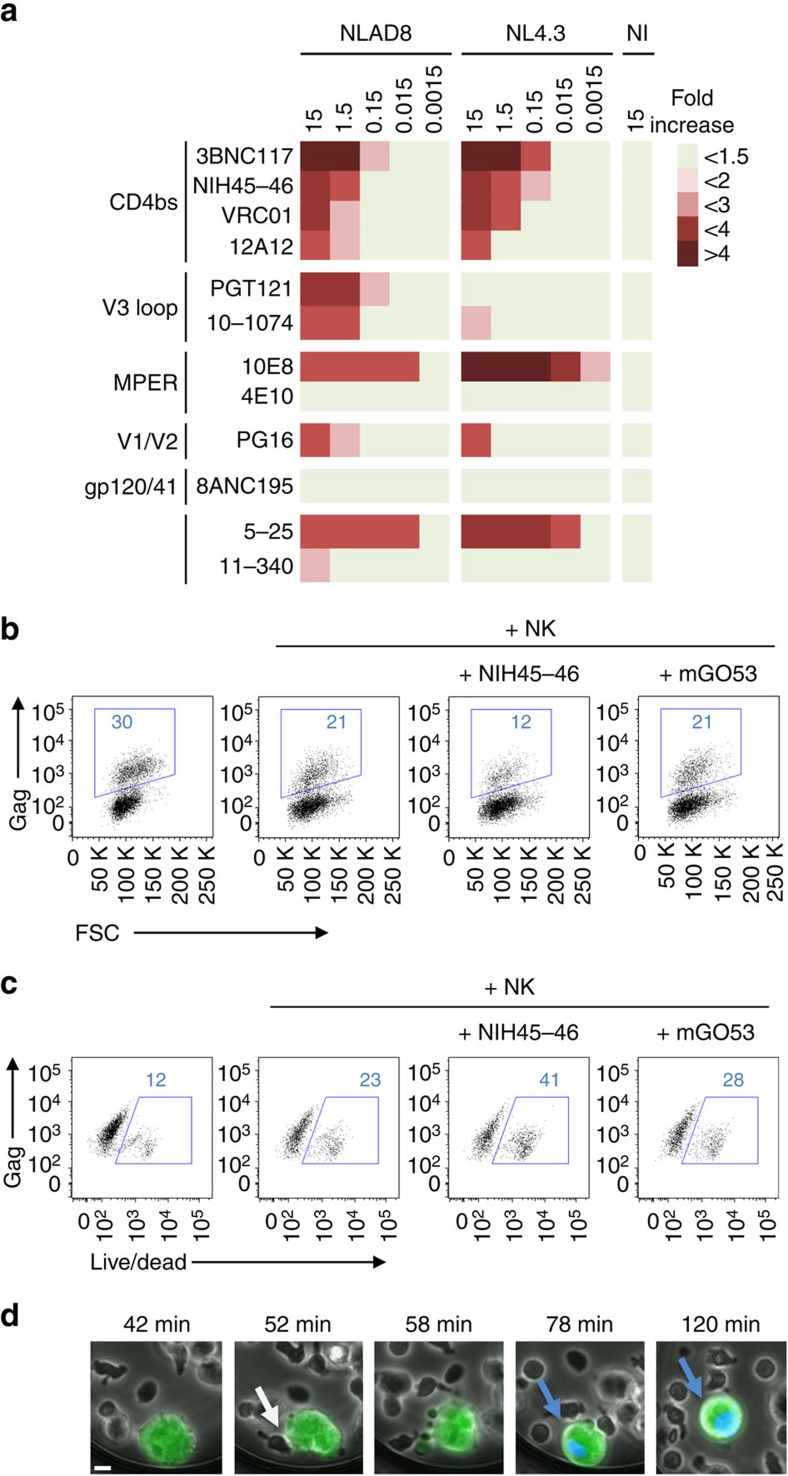
Analysis of the ADCC activity of bNAbs. (**a**) Analysis of the ability of bNAbs bound to infected cells to signal through FcγRIII. MT4C5 cells infected with HIV-1 (NLAD8 or NL4.3 strains) were incubated with the indicated antibodies and with a Jurkat indicator cell line expressing FcγRIII. Upon FcγRIII binding, activation of the NFAT transcription factor induces luciferase. Raw results are presented in Supplementary Fig. 1. The heat map represents the fold increase of the signal over background. (**b**) CEM-NKR cells infected with HIV-1 (NL4.3 strain) were incubated with NIH45–46 bNAb or with the mGO53 isotype antibody and with NK cells. After 4 h, the % of Gag^+^ CEM-NKR target cells (indicated in blue) was measured by flow cytometry. One representative experiment (out of six) is shown. FSC, forward scatter. (**c**) The viability of infected CEM-NK cells was assessed by flow cytometry using the live/dead cell marker. One representative experiment (out of six) is shown. (**d**) CEM-NKR cells infected with NL4–3 encoding IRES-GFP were incubated with NIH45–46 bNAb and plated with primary NK cells. To distinguish dead cells, DAPI dye was added and cells were imaged by time-lapse microscopy. The green cell represents infected live CEM-NKR cells, and turn blue when dying (blue arrow). NK cells are smaller in size. One representative field (corresponding to Supplementary Movie 1) is shown. The arrow indicates a contact between CEM-NKR and NK cells. Scale bar, 2 μm.

We examined the ADCC activity of bNAbs against HIV-1-infected cells. We first investigated the ability of a panel of ten anti-HIV-1 bNAbs to induce signalling through FcγRIII (or CD16). The FcγRIII is the main receptor on NK cells that detects antibody-opsonized targets, and initiates the signalling that leads to ADCC. We previously showed that most of the selected bNAbs neutralize HIV-1 cell-to-cell transmission^7^. These antibodies are IgG1 and contain the same Fc region. They target the CD4-binding site (VRC01, NIH 45–46, 3BNC117, 12A12), the glycan-dependent V1/V2 loops (PG16), the V3 loop (PGT121, 10–1074), the gp120/gp41 interface (8ANC195) and the gp41 membrane-proximal external region (MPER)(10E8 and 4E10)^2^,^3^,^4^,^5^,^28^,^29^. As controls, we added two non-bNAbs antibodies, 5–25 (recognizing the gp41 immuno-dominant epitope) and 11–340 (a cross-neutralizing anti-V3 crown isolated from an elite neutralizer)^30^. To determine how the antibodies bridge HIV-infected cells to FcγRIII-expressing cells, CD4^+^ lymphoid cells (MT4) infected with the prototypic R5-tropic NLAD8 or X4-tropic NL4.3 HIV-1 were incubated with bNAbs before co-culture with Jurkat NFAT-luc FcγRIII cells, which express an NFAT-luciferase reporter activated by FcγRIII stimulation^23^. NLAD8-infected cells induced FcγRIII stimulation with 8 out of 12 antibodies, with variable efficiencies (EC_50_ varying from 0.015 to 4.2 μg ml^−1^ for the active antibodies; Fig. 1a and Supplementary Fig. 1B). Similar results were obtained with NL4.3, with the exception of V3-specific bNAbs, which were poorly active (Fig. 1a and Supplementary Fig. 1A).

We then asked whether FcγRIII signalling was associated with a killing activity of bNAbs. We first assessed the activity of NIH45–46, to determine the optimal conditions of the assay. CEM-NKR cells infected with NLAD8 or NL4.3 were pre-incubated with NIH45–46 before co-culture with NK cells for 4 h. We evaluated the disappearance of Gag^+^ target cells, as readout for ADCC activity (Fig. 1b). A typical experiment showed that the unrelated control antibody mGO53 was inactive, whereas NIH45–46 (at 1.5 μg ml^−1^) induced the disappearance of about 40% of NL4.3-infected cells (Fig. 1b). This disappearance was primarily due to killing, as demonstrated by the presence of dying Gag^+^ cells (Fig. 1c), and by upregulation of the degranulation marker CD107a on NK cells (Supplementary Fig. 1C). Of note, the disappearance of Gag^+^ cells was not due to the neutralization activity of the bNAbs, as no decrease of Gag^+^ cells was observed when NK cells were omitted in the co-culture (Supplementary Fig. 1D). We also visualized the killing of infected cells using time-lapse microscopy. The addition of 4,6-diamidino-2-phenylindole (DAPI), which stains the nucleus of dying cells, allowed us to monitor in real time the fate of cells infected with an IRES-GFP-encoding NL4.3 HIV-1. In the presence of NIH45–46, infected cells rapidly changed morphology and stained for DAPI following interaction with NK cells (see an example Fig. 1d and Supplementary Movie 1). Of note, the non-infected bystander cells present in the co-culture were not killed by the bNAb (Fig. 1d and Supplementary Movie 1).

**Figure 2:**
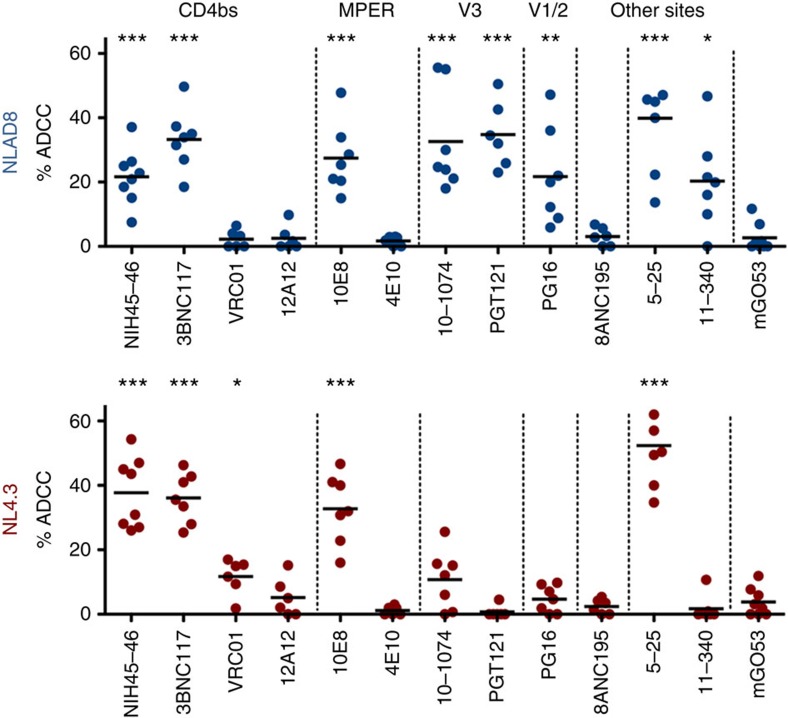
Identification of bNAbs that kill HIV-1-infected lymphocytes. The 12 indicated antibodies were tested at 1.5 μg ml^−1^ on CEM-NKR cells infected with NLAD8 or NL4.3 strains. ADCC was calculated as the disappearance of Gag^+^ cells with or without antibody (*N*=6–8 experiments). Each dot represents a single NK donor. Significance was determined by comparing each antibody to mGO53; ***P<0.001; **P<0.01; *P<0.05, Wilcoxon test).

We next evaluated the ADCC capacity of the full panel of antibodies (Fig. 2). To facilitate comparisons, the antibodies were first used at 1.5 μg ml^−1^. Most of the 12 antibodies (including 6 with NLAD8 and 4 bNAbs with NL4.3) triggered a significant disappearance of infected cells (20–50% decrease of Gag^+^ cells in 4 h). The most active bNAbs corresponded to those which efficiently induced FcγRIII stimulation: NIH45–46 and 3BNC117, which target the CD4bs, the clonally related anti-glycan/V3 antibodies 10–1074 and PGT121, and the MPER targeting 10E8. In contrast, other bNAbs were less active (PG16, VRC01) or inactive (12A12, 4E10 and 8ANC195). The disappearance of Gag-expressing cells required interaction with FcRs, as demonstrated using the L234A-L235A (LALA) mutation, which abrogates FcR binding^9^,^23^. The LALA mutants of five bNAbs maintained their ability to neutralize HIV-1 virions, and hence to bind Env, but lost ADCC potency (Supplementary Fig. 2A). Altogether, there results indicate that only a fraction of the bNAbs induces FcγRIII stimulation and killing of HIV-infected cells.

### Binding of bNAbs at the surface of HIV-1-infected T cells

**Figure 3:**
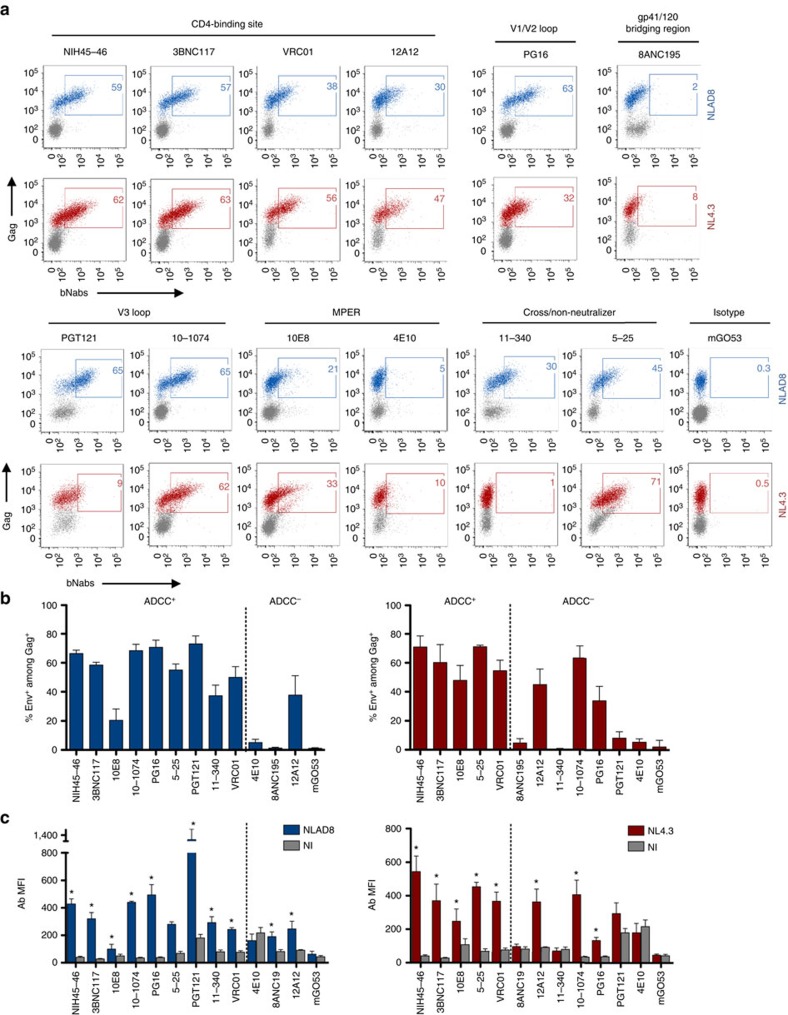
Binding of bNAbs at the surface of HIV-1-infected lymphocytes. (**a**) CEM-NKR cells infected with HIV-1 (NLAD8 or NL4.3) were incubated with the indicated bNAbs (15 μg ml^−1^) at 4 °C and surface levels were analysed by flow cytometry. The numbers indicate the % of bNAb^+^ cells among infected (Gag^+^) cells. One representative experiment (out of six) is shown. The gates were first set on the staining obtained with the mGO53 isotype control. For the bNAbs displaying background staining to the fraction of Gag-negative cells (PGT121 and 8ANC195), the gates were adjusted to decrease this background. (**b**,**c**) The binding of the 12 indicated antibodies to the surface of CEM-NKR cells infected with HIV-1 NLAD8 (blue) or NL4.3 (red) was determined by flow cytometry. The antibodies are classified according to their competence to eliminate (ADCC^+^: >20%) or not (ADCC^−^:<20%) infected cells in the ADCC assay. (**b**) Results and expressed as the % of Env^+^ cells among Gag^+^ cells. (**c**) The median fluorescence intensity (MFI) of staining among Gag^+^ cells is shown (*N*=3 experiments; error bars indicate s.e.m. and significance was determined by comparing stainings to non-infected (NI) cells, *P<0.05, Mann–Whitney test).

To examine the mechanism of ADCC by bNAbs, we assayed their ability to bind HIV-1-infected cells. As previously shown with sera from infected individuals^19^,^22^,^31^, flow cytometry indicated that the bNAbs displaying strong ADCC activity efficiently bound (at 4 °C) HIV-1-infected lymphocytes (Fig. 3a,b and Supplementary Fig. 2B for the gating strategy). The bNAbs primarily bound to Gag^+^ cells and not to bystander cells. Up to 70% of Gag^+^ cells exposed detectable Env epitopes, when the antibodies were used at the highest concentration of 15 μg ml^−1^. The steady-state levels (corresponding to the Median Fluorescence Intensity) varied with each bNAb (Fig. 3c). In contrast, ADCC-inactive bNAbs 4E10 and 8ANC195 did not detectably bind infected cells (Fig. 3). PGT121, which displayed ADCC activity against NLAD8 and not NL4.3, selectively bound NLAD8-infected cells. Of note, with NL4.3, a ‘diagonal' intermediate population, which corresponded to Gag-low cells, was detected with two antibodies (5–25 and 10E8; Fig. 3). This diagonal population was not observed in non-infected cells. It may correspond to cells infected at low levels, and/or to cells which may have recently bound incoming viral particles and expose epitopes recognized by these antibodies.

**Figure 4:**
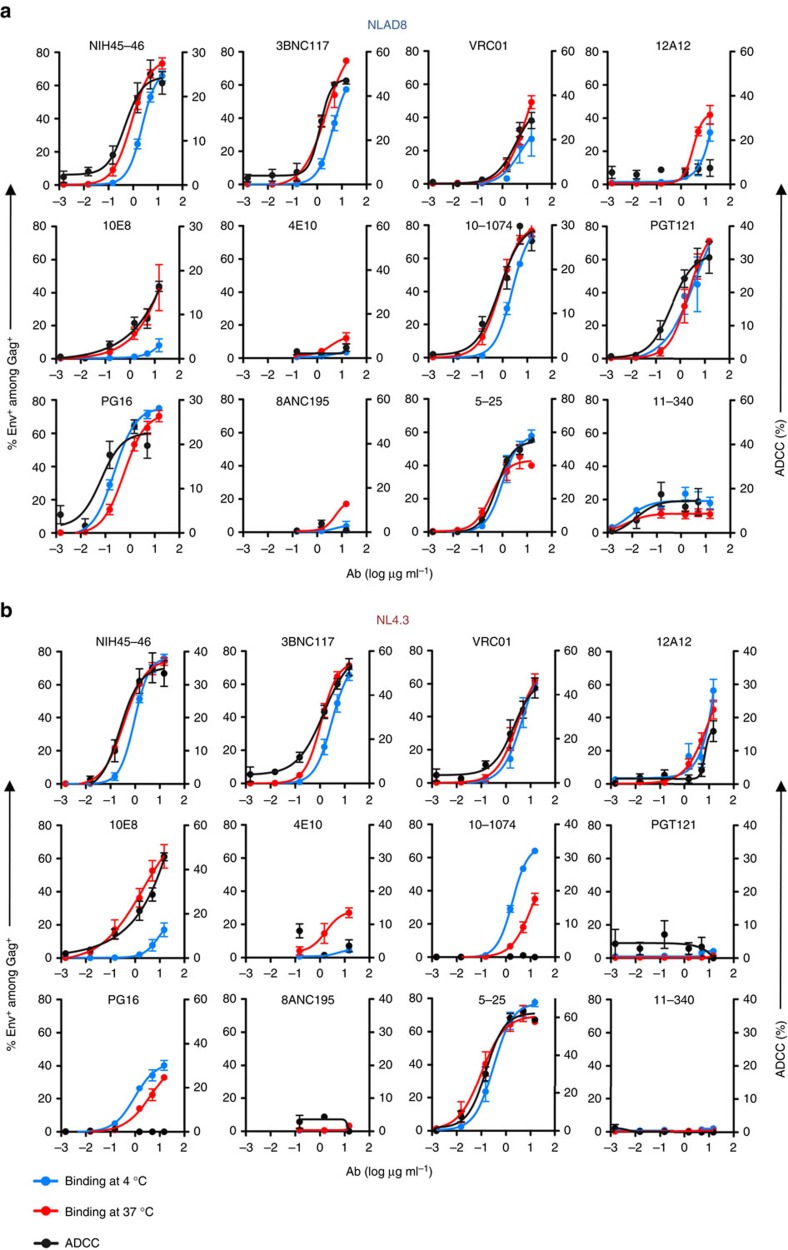
Dose–response analysis of binding of bNAbs and ADCC activity against HIV-1-infected lymphocytes. CEM-NKR cells infected with HIV-1 NLAD8 (**a**) or NL4.3 (**b**) were incubated with the indicated concentrations of antibodies at 4 or 37 °C and surface levels were analysed by flow cytometry. The numbers indicate the % of bNAb^+^ cells among infected (Gag^+^) cells (left axis) and the % of ADCC (right axis). ADCC *y* axes were adjusted for each antibody to facilitate comparisons with the binding profile. For measurement of ADCC, HIV-1-infected CEM-NKR cells were incubated with the indicated antibodies and with NK cells. After 4 h, the % of Gag^+^ CEM-NKR target cells was measured by flow cytometry. The % of ADCC was calculated as the disappearance of Gag^+^ cells (*N*=3 independent experiments for binding; killing assays were performed using at least two NK cell donors; Error bars indicate s.e.m.).

To document the binding and killing activities, we tested the antibodies individually at concentrations varying from 0.015 to 15 μg ml^−1^ (Fig. 4). Binding on CEM-NKR cells infected with NLAD8 or NL4.3 was performed either at 4 °C to assess the steady-state levels of Env epitope exposure, or at 37 °C to reflect the experimental conditions of the ADCC assay. With each antibody, the % of infected cells positive for bNAb binding increased with the concentration and often reached a plateau at 1.5–15 μg ml^−1^ (Fig. 4). As expected, binding was generally more efficient at 37 °C than at 4 °C. This was particularly marked with 10E8, which barely bound infected cells at 4 °C, but displayed significant opsonization at 37 °C. Exceptions were also observed with 10–1074. This antibody-bound NL4.3-infected cells more efficiently at 4 °C than at 37 °C. In contrast, a strong binding occurred at both temperatures with NLAD8-infected cells. The killing activity of the antibodies increased with the concentration and mirrored binding at 37 °C (Fig. 4). A Spearman rank analysis indicated that the two variables were often correlated (Supplementary Table 1). Again, rare discrepancies were detected. 10–1074 potently killed cells infected with NLAD8, and not with NL4.3, despite binding to the later at the highest concentration tested. The same situation was observed with PG16, which poorly bound NL4.3-infected cells but did not display detectable ADCC activity (Fig. 4). Of note, the % of bNAb^+^-infected cells (Fig. 4) mirrored the intensity of binding (MFI), which also increased with the concentration of antibody tested (Supplementary Fig. 3).

**Figure 5:**
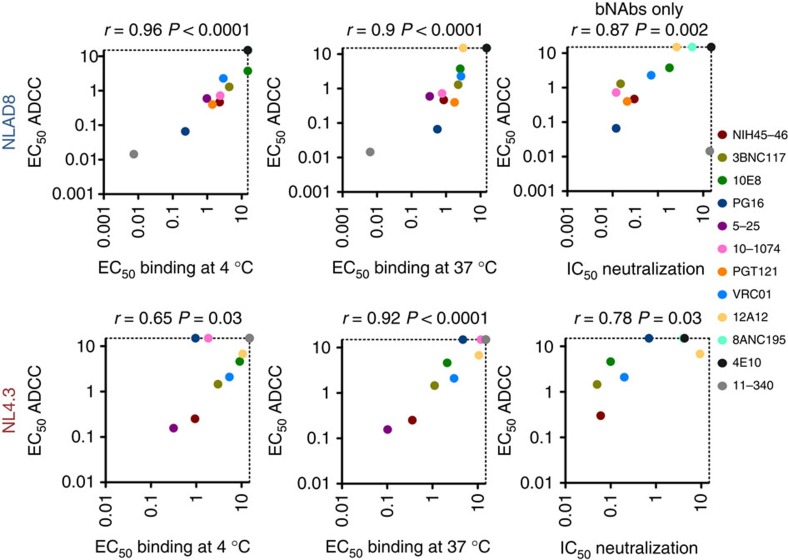
Correlates of ADCC activity of bNAbs. For each antibody, the efficacy of binding and of ADCC against CEM-NKR cells infected with NLAD8 or with NL43 was calculated. The neutralizing activity of the antibodies against cell-free HIV was tested in the TZM-bl assay. EC_50_ (in μg ml^−1^), defined as the effective concentration mediating 50% of the maximal effect, are summarized in Supplementary Table 2. Correlations were analysed by Spearman correlation coefficient (*r*).

A dose–response analysis of the antibody concentration demonstrated that EC_50_, defined as the effective concentration mediating 50% of the maximal effect, were generally similar in the binding (at 37 °C) and killing assays (Fig. 4 and Supplementary Table 2). In both assays, the EC_50_ varied from 0.2 to >15 μg ml^−1^, depending on the antibody (Fig. 4 and Supplementary Table 2). There was a significant correlation between the binding potency of the antibodies, at either 4 or 37 °C and their killing activities against CEM-NKR cells infected with NLAD8 or NL4.3 (Fig. 5). Similarly, the ADCC potency was generally correlated with the neutralizing activity of the antibodies (Fig. 5 and Supplementary Table 1). Notable exceptions include the non-bNAb 5–25, which did not neutralize NLAD8 or NL4.3 but displayed a potent ADCC activity against these viruses.

To visualize the binding of bNAbs on infected cells, we performed immunofluorescent microscopy (Supplementary Fig. 4A) and found a co-localization of NIH45–46 with mature Gag (p17) proteins, whereas immuno-gold staining and scanning electron microscopy (Supplementary Fig. 4B) indicated a preferential binding of the bNAb to viral budding sites.

### Stability of bNAbs at the surface of HIV-1-infected T cells

**Figure 6:**
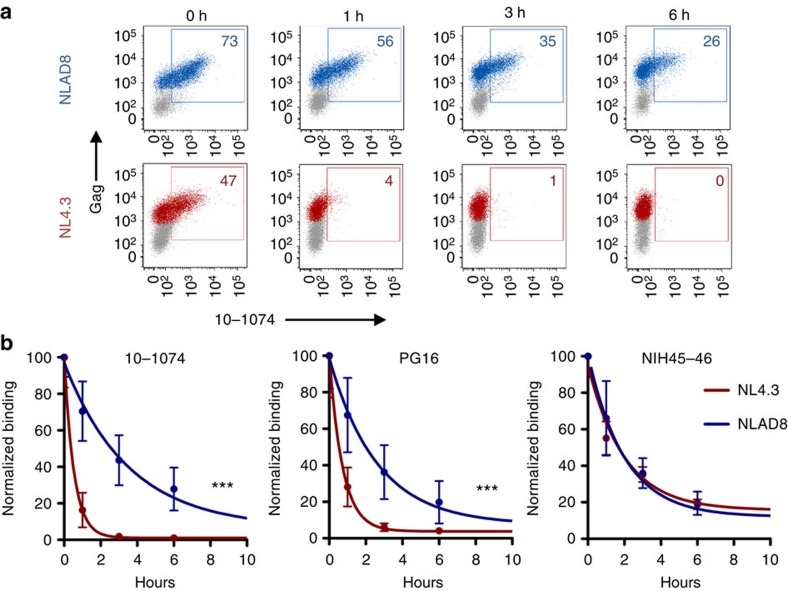
Binding and stability of bNAbs at the surface of HIV-1-infected lymphocytes. (**a**) CEM-NKR cells infected with HIV-1 (NLAD8 or NL4.3) were incubated with 10–1074 bNAb (at 4 °C) and surface levels were analysed by flow cytometry after the indicated incubation times at 37 °C. The numbers indicate the % of bNAb^+^ cells among infected (Gag^+^) cells. One representative experiment (out of six) is shown. (**b**) Decrease of surface stainings of the indicated bNAbs after incubation at 37 °C. (*N*=3–6 experiments; error bars indicate s.e.m. and significance was determined by comparing NLAD8- and NL4.3-infected cells ***P<0.001 extra sum-of-squares F test).

We then measured the cell surface stability of Env-bNAb complexes at 37 °C, an additional parameter that we suspect regulates the sensitivity of infected cells to ADCC. We selected 10–1074 and PG16, which both display an ADCC activity against NLAD8 but not against NL4.3, as well as NIH45–46, which is active against both viruses. Infected cells were stained with each bNAb at 4 °C, extensively washed and the level of remaining complexes was measured at different times at 37 °C (Fig. 6). 10–1074 bound both NLAD8- and NL4.3-infected cells, the latter less efficiently (73 and 47% bNAb^+^ cells at steady-state levels, respectively, Fig. 6a). With NLAD8, the bNAb-Env complex was relatively stable at the cell surface, with a half-life of 2.5 h (Fig. 6a,b). This longevity was strikingly reduced with NL4.3 (half-life of 30 min), consistent with the higher binding of 10–1074 to NL4.3-infected cells at 4 °C than at 37 °C (Fig. 4). Similarly, PG16-Env complexes were less stable with NL4.3 than with NLAD8 (Fig. 6b). In contrast, NIH45–46 remained stably bound with the two viruses (half-life of 2.5 h). These results, as well as those with 3BNC117 and 10E8 (Supplementary Fig. 5A), indicate that an efficient ADCC activity is associated with a sustained presence of Env-bNAb complexes at the cell surface. It is likely that the reduced surface stability of some bNAb-Env complexes results from dissociation of the bNAb due to low affinity. It will be worth exploring whether other mechanisms, including Env endocytosis or shedding of gp120, are also involved in the turnover of surface-bound bNAbs.

To evaluate how the affinity of a bNAb towards Env regulates its binding and killing activity, we selected 10–1074 and the related 10–1369 antibody. They target the same epitope, but displaying a fivefold difference of affinity to YU-2b gp140 trimers (KD of 4 × 10^−9^ and 2 × 10^−8^ M, respectively^29^). As expected, both 10–1074 and 10–1369 neutralized infection with cell-free YU-2b virions (IC_50_ of 1 and 3 μg ml^−1^, respectively, Supplementary Fig. 5B). However, 10–1369 poorly bound to YU-2b-infected cells, and did not trigger ADCC, when compared with 10–1074 (Supplementary Fig. 5B). Thus, results obtained with these two antibodies suggest that efficient killing is associated with a stronger affinity than that necessary for inhibiting cell-free infection.

### Binding and ADCC activity of bNAbs against various HIV-1

**Figure 7:**
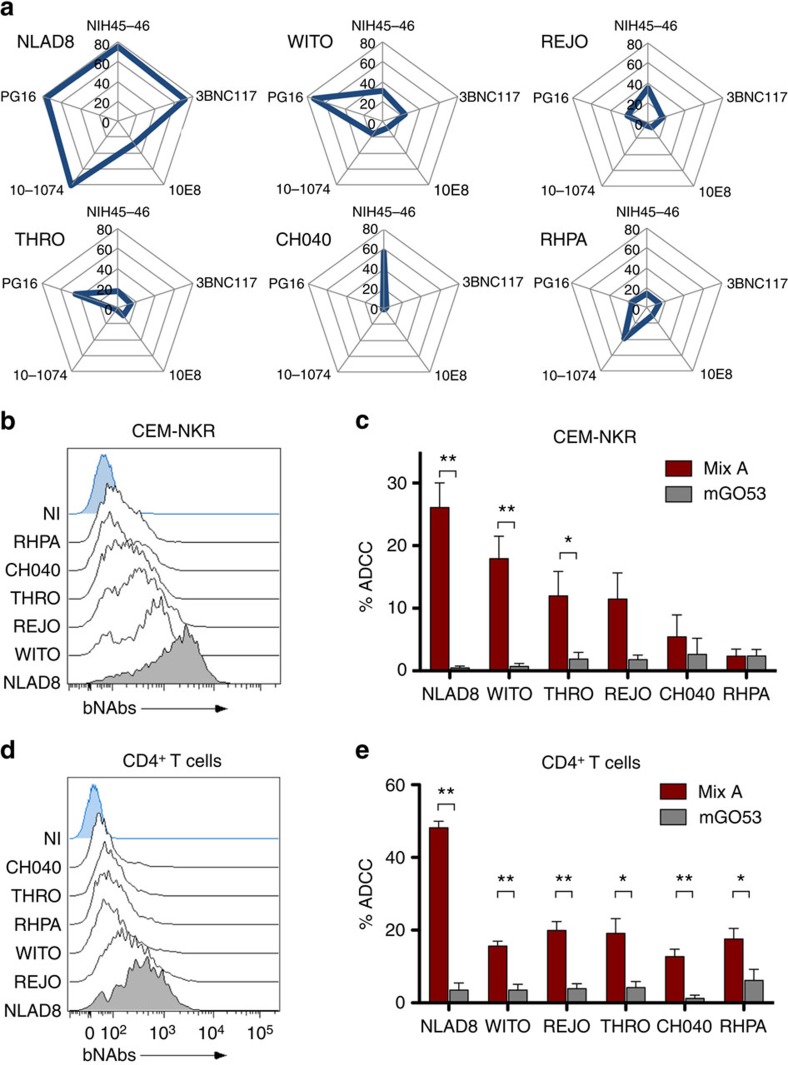
Binding of bNAbs and ADCC activity against lymphocytes infected with various HIV-1 strains. (**a**) CEM-NKR cells infected with the indicated HIV-1 (NLAD8 or five Transmitted/Founder HIV-1) were incubated with the five indicated bNAbs at 4 °C and surface levels were analysed by flow cytometry. The radar plots indicate the % of bNAb^+^ cells among infected (Gag^+^) cells. One representative experiment (out of four) is shown. (**b**) The binding of a combination of the five bNAbs (MixA: NIH45–46; 3BNC117; 10E8; 10–1074 and PG16) to the surface of CEM-NKR cells infected with various HIV-1 (NLAD8 or five T/F HIV-1) was determined by flow cytometry among Gag^+^ cells. One representative experiment (out of four) is shown. (**c**) CEM-NKR cells infected with the indicated HIV-1 strains were incubated with MixA or with mGO53 control antibody and with NK cells. After 6 h, the % of Gag^+^ CEM-NKR target cells was measured by flow cytometry. The % of ADCC was calculated as the disappearance of Gag^+^ cells (*N*=6 experiments; Error bars indicate s.e.m. and significance was determined by comparing MixA to mGO53; **P<0.01; *P<0.05, Wilcoxon test). (**d**) The binding of MixA to the surface of primary CD4^+^ T cells infected with various HIV-1 (NLAD8 or five T/F HIV-1) was determined as in **b**. (**e**) The ADCC activity of MixA against primary CD4^+^ T cells infected with the indicated HIV-1 strains was determined as in **c**.

Primary HIV-1 isolates, including Transmitted/Founder (T/F) viruses may be less sensitive to neutralization by bNAbs than laboratory-adapted strains^32^,^33^. To explore the sensitivity of primary HIV-1 to ADCC, we first measured the exposure of Env epitopes at the surface of CEM-NKR cells infected with five T/F strains (WITO, THRO, REJO, CH077, RHPA)^32^, using five bNAbs (Fig. 7a). The T/F viruses were selected based on their ability to efficiently replicate *in vitro*. Levels of accessible Env epitopes were lower with T/F viruses than with NLAD8. There was a strong variability in the pattern of bNAb binding. In contrast to NLAD8, which was recognized by all five bNAbs, cells infected with T/F viruses generally bound only 1–3 antibodies (Fig. 7a). There was no single bNAb recognizing all T/F viruses. We thus mixed the five bNAbs (MixA; NIH45–46, 3BNC117, 10–1074, PG16 and 10E8, each at 1.5 μg ml^−1^) and analysed the ability of MixA to bind infected cells and to perform ADCC. MixA efficiently bound cells infected with NLAD8 and, to a lower extent, with T/F viruses (Fig. 7b), reflecting the profile observed with individual bNAbs. Interestingly, MixA displayed an ADCC activity against cells infected with some, but not all T/F viruses (Fig. 7c). The killing activity against WITO, REJO and THRO was less potent than with NLAD8, correlating with opsonization levels. CH040 and RHPA-infected cells, which expose lower levels of Env epitopes, were barely sensitive to killing by MixA (Fig. 7c).

MixA-bound primary CD4^+^ T cells infected with T/F with variable intensities (Fig. 7d). Primary lymphocytes infected with NLAD8, and to a lower extent with T/F viruses, were killed by MixA (Fig. 7e). Thus, T/F HIV-1 isolates poorly expose Env epitopes at the surface of infected lymphocytes. When combined, bNAbs act in a complementary manner to bind infected cells, providing increased ADCC breadth against primary HIV-1 isolates. These results are in line with experiments demonstrating that bNAb combinations control HIV-1 replication in humanized mice^27^, and block cell–cell transmission of T/F in culture^34^.

### Activity of bNAbs against reactivated HIV-1-infected cells

**Figure 8:**
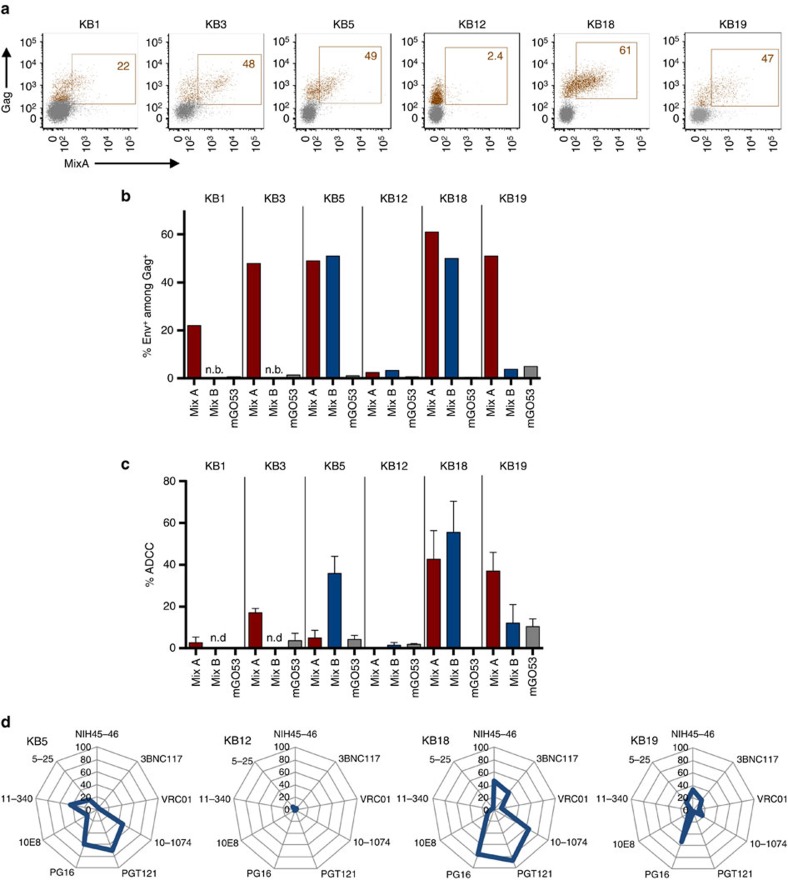
Binding of bNAbs and ADCC activity against reactivated HIV-1-infected cells from the viral reservoir in patients on HAART. (**a**) Purified CD4^+^ T cells from the six indicated patients on HAART were activated and viral replication was followed by flow cytometry. When the % of Gag^+^ cells was >5%, cells were stained with the MixA bNAb combination at 4 °C. The figures indicate the % of bNAb^+^ cells among Gag^+^ cells. One representative experiment (out of two or three for each patient) is shown. (**b**) The binding of two combinations of antibodies (MixA, red columns) and MixB (blue columns) to cells from the same patients is shown. The figures indicate the % of bNAb^+^ cells among Gag^+^ cells. One representative experiment (out of two or three for each patient) is shown. (**c**) The ADCC activity of MixA and MixB against cells from the indicated patients is shown. Target cells were used for ADCC experiments when the fraction of Gag^+^ cells was above 5%. (*N*=2–4 replicates with different NK cells; Error bars indicate s.e.m.). (**d**) The binding of individual antibodies (at 4 °C) to cells from the indicated patients is shown. The figures indicate the % of bNAb^+^ cells among Gag^+^ cells. The radar plots indicate the % of bNAb^+^ cells among infected (Gag^+^) cells. One representative experiment (out of two or three for each patient) is shown.

We then asked whether bNAbs could target HIV-1 produced after stimulation of lymphocytes isolated directly from patients, as bNAbs are potential therapeutic molecules that may reach the reactivated viral reservoir in HIV-1-infected individuals. We selected infected individuals under suppressive antiretroviral treatment (viral loads <40 copies per ml, see Supplementary Table 3 for details). We used a viral outgrowth assay, in which phytohaemagglutinin (PHA) treatment activates resting CD4^+^ T cells and induces HIV-1 spread from latently infected cells^35^. HIV-1 Gag^+^ cells started to be detected by flow cytometry at days 7–12 post reactivation in 6 individuals (out of 20 tested, Supplementary Fig. 6A) and increased over time, indicating that reactivated viruses were infectious. Cell surface Env expression was assessed with MixA or, for some of the patients, with MixB, a second cocktail of antibodies including VRC01, PGT121, 5–25 and 11–340 (see Supplementary Fig. 6B for the gating strategy). Binding with either MixA or MixB was observed with variable intensities on reactivated cells from five out of the six individuals (Fig. 8a,b and Supplementary Fig. 6C,D). In one individual (KB12), despite ongoing viral replication, no Env signal was detected with either Mix. We then tested the sensitivity of reactivated cells to ADCC, using the two bNAb combinations (Fig. 8c and Supplementary Fig. 6E). Interestingly, in cells from four out of the five patients that bound the antibodies, an ADDC activity was detected with either MixA or MixB, leading to the disappearance of 10–50% of Gag^+^ cells. Thus, there is a strong heterogeneity in the levels of Env epitopes expressed at the surface of reactivated cells, which is associated with variable susceptibility to ADCC.

Env epitope exposure was further assessed by sampling reactivated cells from four donors with individual bNAbs present in the two cocktails (Fig. 8d). As expected, no single antibody bound to reactivated KB12 cells. With KB5, KB18 and KB19 samples, three to six bNAbs out of the nine tested displayed significant attachment to reactivated cells, each patient displaying a different binding profile.

## Discussion

We have analysed here the ADCC activity of bNAbs against laboratory-adapted, transmitted/founder and reactivated HIV-1 derived from the viral reservoir. We report that a subset of bNAbs effectively kills HIV-1-infected lymphocytes. All antibodies tested contain the same Fc region, implying that the differences in their ability to signal through FcγRIII and mediate ADCC is dependent on their variable regions. Thus, differences in killing efficiency are likely due to changes in binding of the antibody and accessibility of the Fc region when bound to infected cells. Env steady-state levels and surface stability of bNAbs regulate their ADDC potency. We show a significant correlation between, the intensity of antibody binding at the cell surface, the stability of this binding at 37 °C, neutralization activity and their capacity to eliminate HIV-1-infected cells. By using the related antibodies 10–1074 and 10–1369, we further demonstrate a link between the affinity of a given antibody to the Env trimer and its ADCC potency. Our results extend previous work showing that antibody affinity is related to neutralizing activity^36^ and provide a comprehensive mechanistic analysis of the ADCC activity of a panel of the newest bNAbs. We show that Env epitope exposure on infected cells is highly variable, depending on the viral isolate. Future work will help assess the role of viral proteins and other factors in the modulation of epitope exposure and ADCC. PHA-activated CD4^+^ T lymphocytes derived from patients' cells expose sufficient amounts of Env epitopes required for recognition by combinations of bNAbs and killing by NK cells. Our experiments offer a mechanistic explanation as to how bNAbs associated with viral inducers decrease rebound from latent reservoirs in humanized mice^27^. We further show that induced viral reservoirs display an extreme heterogeneity in Env epitope exposure. This reflects the variable sensitivity of virions from the reservoir to neutralization by bNAbs^37^. It would be worthwhile to follow the landscape of epitopes longitudinally, in order to determine whether reactivated founder viruses evolve over time, and originates from clones or populations of cells^38^,^39^ with homogeneous or heterogeneous Env profiles. In HIV-1-infected individuals, low-level viraemia during effective highly active antiretroviral therapy likely result from expression of archival virus and covert viral replication^40^. Latent proviruses are found predominantly in subsets of resting memory cells, which are largely non-permissive for viral gene expression^35^,^41^,^42^. It will be of interest determining whether bNAbs may kill with similar efficiencies cells from the latent reservoir, or from a population of lymphocytes with ongoing low-level of viral replication. This may be achieved by sorting resting memory T cells from patients and performing ADCC assays with various cell activators or latency-reversing agents^35^. It will also be of interest to assess whether the bNAbs may kill the actual reactivated latently infected cells before the virus spreads to neighbouring cells. This could be performed by visualizing killing of infected cells after viral reactivation in humanized mouse models.

Our results directly demonstrate that bNAbs, in addition to other immune interventions^43^,^44^, represent an efficient tool for consideration in ‘shock and kill' strategies aimed at purging the viral reservoir^14^,^26^. A bNAb-based HIV-1 cure will likely require a personalized screening of the pattern of Env epitope exposure on reactivated cells, to determine the optimal combination of antibodies. The reported data on differences among bNAbs in their ability to kill HIV-1-infected lymphocytes will enable a better understanding of the functional attributes of antibodies for prevention and cure strategies.

## Methods

### Cells and viruses

CEM-NKR-CCR5 cells (referred to as CEM-NKR) obtained from the NIH AIDS reagent programme. MT4C5 cells were derived from MT4 cells obtained from ATCC to express CCR5 (ref. 45). Primary CD4^+^ T cells and NK cells were purified from peripheral blood of healthy human donors from the Etablissement Français du Sang (EFS) in accordance with EFS ethical guidelines by density gradient centrifugation followed by immunomagnetic selection (Miltenyi). Purity was 90–98% for each population. After purification, NK cells were CD16^+^, CD32^−^ and CD64^−^. For activation, primary T cells were treated with PHA (1 μg ml^−1^) for 24 h at 37 °C and then cultured in IL-2-containing medium (50 IU ml^−1^) for 3–5 days before use. Virus stocks were prepared by transfection of 293T cells, along with VSV-G to normalize infectivity^45^. Cells were infected with HIV-1 strains NL4.3, NLAD8, YU-2b and Transmitted/Founder (CH040, RHPA, THRO, REJO and WITO; obtained from the NIH AIDS reagent programme)^45^,^46^. Briefly, viral inocula (0.5–5 ng of p24/10^6^ cells for MT4C5 and CEM-NKR cells, 50–100 ng of p24/10^6^ cells for primary CD4^+^ T cells) were adjusted to achieve similar levels of Gag^+^ cells (around 50% in CEM-NKR cells and 15–30% in primary CD4^+^ T cells) at 48 h post infection.

### Antibodies

All anti-Env antibodies and bNAbs, as well as the isotype control mGO53, were produced as recombinant monoclonal antibodies carrying the same human IgG1 Fc region by co-transfection of 293T or 293F cells^29^. Antibodies were purified by batch/gravity-flow affinity chromatography using protein G sepharose 4 fast flow beads (GE Healthcare). Absence of protein contaminations and antibody aggregations were checked using in-gel protein Silver staining and dynamic light scattering (DynaPro plate reader, Wyatt), respectively. All IgG preparations were verified to be endotoxin free.

### FcγRIII stimulation assay

Activation of FcγRIII signalling was measured by using a Jurkat NFAT-luc FcγRIII cell line (Promega) following the manufacturer's recommendations. FcγRIII signalling activates the NFAT transcription factor, inducing expression of firefly luciferase^23^. HIV-1-infected MT4C5 cells were co-cultivated with Jurkat NFAT-luc FcγRIII (ratio 1:1) for 18 h at 37 °C. Cells were then lysed and luciferase was measured on an Enspire Plate reader (Perkin-Elmer).

### ADCC assay

HIV-1-infected target CEM-NKR or primary CD4 T cells were stained using the Far Red DDAO cell tracker (Life technologies). 2–5 × 10^4^ targets were plated in U-bottom 96-well plates and incubated with antibodies (1.5 μg ml^−1^ unless otherwise stated) for 5 min at room temperature. NK cells were added in each well (at a ratio of 1 CEM-NKR:10 NK or 1 primary CD4 T cell:1 NK, respectively). Plates were spun 1 min at 300*g* to promote cell contacts and incubated at 37 °C for 4 h (for primary CD4 T cells) or 6 h (for CEM-NKR cells). Cells were then stained for intra-cellular Gag with the anti-Gag KC57 murine monoclonal antibody^45^. In the indicated experiments, an anti-CD107a antibody (clone H4A3, BD Biosciences, final dilution of 1:50) was added in the cell co-culture to assess NK degranulation. To measure cell viability, the live/dead fixable aqua dead cell marker (1: 1,000 in PBS, Life technologies) was added 20 min at 4 °C before fixation. Data were acquired on a BD FACS CANTO II and analysed using FlowJo software. The frequencies of Gag^+^ cells among Far-Red^+^ cells were determined. ADCC was calculated using the following formula: 100 × (% of Gag^+^ target cells plus NK without antibody—% of Gag^+^ target cells plus effector with antibody)/(% of Gag^+^ target cells plus NK without antibody). Negative values were set to zero. The maximum values obtained in the ADCC assay was a disappearance of ∼60% of Gag^+^ cells.

### Binding and stability of bNAbs at the cell surface

Cells (0.5–2 × 10^4^ per well) were incubated 1 h at 4 °C or, when stated, at 37 °C with anti-Env bNAbs or with an isotype human IgG1 control (mG053) at 15 μg ml^−1^ (unless otherwise stated) diluted in culture medium. Cells were then washed and incubated 30 min at 4 °C with an anti-human IgG1 (H+L) Alexa Fluor 647 (1:400 dilution, Life technologies). Cells were then fixed with 4% paraformaldehyde and processed for intracellular Gag staining. To measure the stability of Env-bNAb complexes at the surface, cells were incubated 1 h at room temperature with bNAbs (15 μg ml^−1^) washed three times with PBS to remove unbound bNAbs and re-suspended in warm culture medium. After the indicated times at 37 °C, the levels of cell-associated bNAbs were revealed using an anti-human IgG1 (H+L) Alexa Fluor 647 (1:400, Life technologies) for 30 min at 4 °C. Cells were then fixed with 4% paraformaldehyde and processed for intracellular Gag staining.

### Neutralization assay

Neutralization of cell-free HIV-1 was measured using TZM-bl cells^7^, which HeLa CD4^+^CCR5^+^ cells carrying an HIV-1 LTR–βgal reporter cassette. One day before infection, 7 × 10^3^ cells were plated in 96-well plates. Cells were infected in triplicate with 1 or 5 ng Gag p24. Viruses were incubated with the indicated bNAbs for 1 h before infection. After 36 h, cells were lysed in PBS, 0.1% NP-40 and 5 mM MgCl_2_ and incubated with the β-gal substrate CPRG (Roche), before measurement of 570-nm optical density. Dose–response inhibition curves were drawn by fitting data to sigmoid dose–response curves (variable slope) using GraphPad Prism software. The % of inhibition was defined as (% signal in non-treated target cells−% signal in bNAb-treated cells)/(% signal in non-treated target cells) × 100. The 50% inhibitory dose (IC_50_) was calculated with GraphPad Prism.

### Confocal microscopy and scanning electron microscopy

Confocal microscopy analysis was performed as described^45^. The following antibodies were used: Anti-Env NIH45–46 bNAb or isotype control (15 μg ml^−1^); anti-Gag p17 (mouse anti-p17 ARP342, Programme EVA Centre for AIDS Reagents, 1:100 dilution) and anti-Gag-FITC (KC57, 1:50 dilution). Acquisitions were performed on a Zeiss LSM700 using a × 63 objective. Images were analysed using FIJI software and assembled with the Magic Montage ImageJ plugin. Immunogold staining and scanning electron microscopy were performed as described^45^. The following antibody was used: Anti-Env NIH45–46 bNAb or isotype controls (150 μg ml^−1^). The images were acquired with a JEOL JSM 6700F field emission scanning electron microscope.

### Live imaging

CEM-NKR cells were infected with the NL4.3-IRES-GFP virus^45^. Cellular compartmentalization in microwells was achieved using 100 μm micromesh array (Microsurfaces) stick on μ-dish (Ibidi) according to the manufacturer instruction. To fill the wells, 0.5–1.5 × 10^3^ CEM-NKR cells were seeded; the dish was then spun for 3 min at 200 g. NIH45–46 bNAb (2 μg ml^−1^) and DAPI (to visualize dying cells) and then 0.5–1 × 10^4^ NK cells were added. The dish was transferred into a Biostation IMQ (Nikon). Ten to fifteen fields were acquired with images taken every 2 min. Image analysis was performed using ImageJ (FIJI). One representative movie was selected.

### Reactivation of HIV-1 from highly active antiretroviral therapy (HAART)-treated patients

All patients were under successful HAART (see Supplementary Table 3 for details). Each participant provided a written consent to participate to the study, which was approved by the regional investigational review board (Comité de Protection des Personnes Ile-de-France VII (Paris, France) and performed according to the European guidelines and the Declaration of Helsinki. For each patient, 50 ml of blood were collected in the presence of EDTA. Peripheral blood mononuclear cells (PBMCs) were isolated by ficoll gradient purification and CD4 T cells were purified as described above. For the Viral Outgrowth Assay, CD4 T cells were stimulated with PHA-M (2 mg ml^−1^, Sigma-Aldrich) in 2.5 ml of culture medium with 100 UI ml^−1^ of IL-2 (R&D) in a 12-well plate. After 24 h, cells were washed to remove PHA-M and resuspended in 2.5 ml of medium containing IL-2. Every 1–2 days, 1 ml of supernatant was harvested and replaced with fresh medium. At the indicated time points, cells were evaluated for Gag expression and bNAb binding by flow cytometry. Cells were used for ADCC experiments when the fraction of Gag^+^ cells was above 5%.

### Data processing and statistical analysis

Calculations were performed and figures were drawn using Excel 2011 or GraphPad Prism 5.0. Statistical analysis was performed using GraphPad Prism, with Wilcoxon matched paired *t*-tests, Mann–Withney unpaired *t*-tests or extra sum-of-squares F test. Spearman correlation coefficients (*r*) were calculated using GraphPad Prism.

### Sample size

The size of the samples was chosen to allow a statistical analysis of the results.

## Additional information

**How to cite this article:** Bruel, T. *et al*. Elimination of HIV-1-infected cells by broadly neutralizing antibodies. *Nat. Commun.* 7:10844 doi: 10.1038/ncomms10844 (2016).

## Supplementary Material

Supplementary Information Supplementary Figures 1-6 and Supplementary Tables 1-3

Supplementary Movie 1 Real time imaging of dying infected T cells in the presence of bNAb and NK cells. CEM-NKR cells infected with NL4-3 encoding IRES-GFP were incubated with NIH45-46 bNAb and plated with primary NK cells. To distinguish dead cells, DAPI dye was added and cells were imaged by time-lapse microscopy. The green cell represents infected live CEM-NKR cells, and turn blue when dying (blue arrow). NK cells are smaller in size. Images were taken every 2 min. One representative movie is shown. The white arrow indicates a contact between CEM-NKR and NK cells.

## References

[b1] Stamatatos L., Morris L., Burton D. R. & Mascola J. R. Neutralizing antibodies generated during natural HIV-1 infection: good news for an HIV-1 vaccine? Nature Med. 15, 866–870 (2009). 19525964 10.1038/nm.1949

[b2] McCoy L. & Weiss R. Neutralizing antibodies to HIV-1 induced by immunization. J. Exp. Med. 210, 209–223 (2013). 23401570 10.1084/jem.20121827 PMC3570100

[b3] Mouquet H. Antibody B cell responses in HIV-1 infection. Trends Immunol. 35, 549–561 (2014). 25240985 10.1016/j.it.2014.08.007

[b4] Klein F. . Antibodies in HIV-1 vaccine development and therapy. Science 341, 1199–1204 (2013). 24031012 10.1126/science.1241144 PMC3970325

[b5] Burton D. R. & Mascola J. R. Antibody responses to envelope glycoproteins in HIV-1 infection. Nat. Immunol. 16, 571–576 (2015). 25988889 10.1038/ni.3158 PMC4834917

[b6] Caskey M. . Viraemia suppressed in HIV-1-infected humans by broadly neutralizing antibody 3BNC117. Nature 522, 487–491 (2015). 25855300 10.1038/nature14411 PMC4890714

[b7] Malbec M. . Broadly neutralizing antibodies that inhibit HIV-1 cell to cell transmission. J. Exp. Med. 210, 2813–2821 (2013). 24277152 10.1084/jem.20131244 PMC3865481

[b8] Reh L. . Capacity of broadly neutralizing antibodies to inhibit HIV-1 cell-cell transmission is strain- and epitope-dependent. PLoS Pathog. 11, e1004966 (2015). 26158270 10.1371/journal.ppat.1004966 PMC4497647

[b9] Hessell A. J. . Fc receptor but not complement binding is important in antibody protection against HIV. Nature 449, 101–104 (2007). 17805298 10.1038/nature06106

[b10] Bournazos S. . Broadly neutralizing anti-HIV-1 antibodies require Fc effector functions for in vivo activity. Cell 158, 1243–1253 (2014). 25215485 10.1016/j.cell.2014.08.023 PMC4167398

[b11] Ko S. Y. . Enhanced neonatal Fc receptor function improves protection against primate SHIV infection. Nature 514, 642–645 (2014). 25119033 10.1038/nature13612 PMC4433741

[b12] Kramski M., Stratov I. & Kent S. J. The role of HIV-specific antibody-dependent cellular cytotoxicity in HIV prevention and the influence of the HIV-1 Vpu protein. AIDS 29, 137–144 (2015). 25396265 10.1097/QAD.0000000000000523

[b13] Euler Z. & Alter G. Exploring the potential of monoclonal antibody therapeutics for HIV-1 eradication. AIDS Res. Hum. Retroviruses 31, 13–24 (2015). 25385703 10.1089/aid.2014.0235 PMC4287163

[b14] Lee W., Parsons M., Kent S. & Lichtfuss M. Can HIV-1-specific ADCC assist the clearance of reactivated latently infected cells? Front. Immunol. 6, 265 (2015). 26074924 10.3389/fimmu.2015.00265 PMC4445400

[b15] Haynes B. F. . Immune-correlates analysis of an HIV-1 vaccine efficacy trial. N. Engl. J. Med. 366, 1275–1286 (2012). 22475592 10.1056/NEJMoa1113425 PMC3371689

[b16] Chung A. W. . Dissecting polyclonal vaccine-induced humoral immunity against HIV using systems serology. Cell 163, 988–998 (2015). 26544943 10.1016/j.cell.2015.10.027 PMC5490491

[b17] Barouch D. H. . HIV-1 vaccines. Protective efficacy of adenovirus/protein vaccines against SIV challenges in rhesus monkeys. Science 349, 320–324 (2015). 26138104 10.1126/science.aab3886 PMC4653134

[b18] Lambotte O. . Heterogeneous neutralizing antibody and antibody-dependent cell cytotoxicity responses in HIV-1 elite controllers. AIDS 23, 897–906 (2009). 19414990 10.1097/QAD.0b013e328329f97d PMC3652655

[b19] Smalls-Mantey A. . Antibody-dependent cellular cytotoxicity against primary HIV-infected CD4+ T cells is directly associated with the magnitude of surface IgG binding. J. Virol. 86, 8672–8680 (2012). 22674985 10.1128/JVI.00287-12 PMC3421757

[b20] Milligan C., Richardson B. A., John-Stewart G., Nduati R. & Overbaugh J. Passively acquired antibody-dependent cellular cytotoxicity (ADCC) activity in HIV-infected infants is associated with reduced mortality. Cell Host Microbe 17, 500–506 (2015). 25856755 10.1016/j.chom.2015.03.002 PMC4392343

[b21] Chung A. W. . Immune escape from HIV-specific antibody-dependent cellular cytotoxicity (ADCC) pressure. Proc. Natl Acad. Sci. USA 108, 7505–7510 (2011). 21502492 10.1073/pnas.1016048108 PMC3088575

[b22] Richard J. . CD4 mimetics sensitize HIV-1-infected cells to ADCC. Proc. Natl Acad. Sci. USA 112, E2687–E2694 (2015). 25941367 10.1073/pnas.1506755112 PMC4443331

[b23] Alvarez R. A. . HIV-1 Vpu antagonism of tetherin inhibits antibody-dependent cellular cytotoxic responses by natural killer cells. J. Virol. 88, 6031–6046 (2014). 24623433 10.1128/JVI.00449-14 PMC4093850

[b24] Arias J. F. . Tetherin antagonism by Vpu protects HIV-infected cells from antibody-dependent cell-mediated cytotoxicity. Proc. Natl Acad. Sci. USA 111, 6425–6430 (2014). 24733916 10.1073/pnas.1321507111 PMC4035966

[b25] Pham T. N., Lukhele S., Hajjar F., Routy J.-P. P. & Cohen É. A. A. HIV Nef and Vpu protect HIV-infected CD4+ T cells from antibody-mediated cell lysis through down-modulation of CD4 and BST2. Retrovirology 11, 15 (2014). 24498878 10.1186/1742-4690-11-15 PMC3930549

[b26] Deeks S. G. . Towards an HIV cure: a global scientific strategy. Nat. Rev. Immunol. 12, 607–614 (2012). 22814509 10.1038/nri3262 PMC3595991

[b27] Halper-Stromberg A. . Broadly neutralizing antibodies and viral inducers decrease rebound from HIV-1 latent reservoirs in humanized mice. Cell 158, 989–999 (2014). 25131989 10.1016/j.cell.2014.07.043 PMC4163911

[b28] Sanders R. W. . HIV-1 VACCINES. HIV-1 neutralizing antibodies induced by native-like envelope trimers. Science (New York, N.Y.) 349, aac4223 (2015). 10.1126/science.aac4223 PMC4498988 26089353

[b29] Mouquet H. . Complex-type N-glycan recognition by potent broadly neutralizing HIV antibodies. Proc. Natl Acad. Sci. USA 109, E3268–E3277 (2012). 23115339 10.1073/pnas.1217207109 PMC3511153

[b30] Mouquet H. . Memory B cell antibodies to HIV-1 gp140 cloned from individuals infected with clade A and B viruses. PLoS ONE 6, e24078 (2011). 21931643 10.1371/journal.pone.0024078 PMC3169578

[b31] Lee W. . Antibody-dependent cellular cytotoxicity against reactivated HIV-1-infected cells. J. Virol. 90, 2021–2030 (2015). 26656700 10.1128/JVI.02717-15 PMC4733999

[b32] Salazar-Gonzalez J. . Genetic identity, biological phenotype, and evolutionary pathways of transmitted/founder viruses in acute and early HIV-1 infection. J. Exp. Med. 206, 1273–1289 (2009). 19487424 10.1084/jem.20090378 PMC2715054

[b33] Seaman M. S. . Tiered categorization of a diverse panel of HIV-1 Env pseudoviruses for assessment of neutralizing antibodies. J. Virol. 84, 1439–1452 (2010). 19939925 10.1128/JVI.02108-09 PMC2812321

[b34] Kong R. . Improving neutralization potency and breadth by combining broadly reactive HIV-1 antibodies targeting major neutralization epitopes. J. Virol. 89, 2659–2671 (2015). 25520506 10.1128/JVI.03136-14 PMC4325730

[b35] Bruner K. M., Hosmane N. N. & Siliciano R. F. Towards an HIV-1 cure: measuring the latent reservoir. Trends Microbiol. 23, 192–203 (2015). 25747663 10.1016/j.tim.2015.01.013 PMC4386620

[b36] Scheid J. F. . Sequence and structural convergence of broad and potent HIV antibodies that mimic CD4 binding. Science 333, 1633–1637 (2011). 21764753 10.1126/science.1207227 PMC3351836

[b37] Chun T.-W. W. . Broadly neutralizing antibodies suppress HIV in the persistent viral reservoir. Proc. Natl Acad. Sci. USA 111, 13151–13156 (2014). 25157148 10.1073/pnas.1414148111 PMC4246957

[b38] Maldarelli F. . Specific HIV integration sites are linked to clonal expansion and persistence of infected cells. Science 345, 179–183 (2014). 24968937 10.1126/science.1254194 PMC4262401

[b39] Cohn L. B. . HIV-1 integration landscape during latent and active infection. Cell 160, 420–432 (2015). 25635456 10.1016/j.cell.2015.01.020 PMC4371550

[b40] Tobin N. H. . Evidence that low-level viremias during effective highly active antiretroviral therapy result from two processes: expression of archival virus and replication of virus. J. Virol. 79, 9625–9634 (2005). 16014925 10.1128/JVI.79.15.9625-9634.2005 PMC1181593

[b41] Chomont N. . HIV reservoir size and persistence are driven by T cell survival and homeostatic proliferation. Nature Med. 15, 893–900 (2009). 19543283 10.1038/nm.1972 PMC2859814

[b42] Ruelas D. S. & Greene W. C. An integrated overview of HIV-1 latency. Cell 155, 519–529 (2013). 24243012 10.1016/j.cell.2013.09.044 PMC4361081

[b43] Shan L. . Stimulation of HIV-1-specific cytolytic T lymphocytes facilitates elimination of latent viral reservoir after virus reactivation. Immunity 36, 491–501 (2012). 22406268 10.1016/j.immuni.2012.01.014 PMC3501645

[b44] Pegu A. . Activation and lysis of human CD4 cells latently infected with HIV-1. Nat. Commun. 6, 8447 (2015). 26485194 10.1038/ncomms9447 PMC4633990

[b45] Casartelli N. . Tetherin restricts productive HIV-1 cell-to-cell transmission. PLoS Pathog. 6, e1000955 (2010). 20585562 10.1371/journal.ppat.1000955 PMC2887479

[b46] Ochsenbauer C. . Generation of transmitted/founder HIV-1 infectious molecular clones and characterization of their replication capacity in CD4 T lymphocytes and monocyte-derived macrophages. J. Virol. 86, 2715–2728 (2012). 22190722 10.1128/JVI.06157-11 PMC3302286

