# Supplementary material for: Elimination of HIV-1-infected cells by broadly neutralizing antibodies
Source: Nat Commun. 2016 Mar 3;7:10844. doi: 10.1038/ncomms10844 (PMC4782064; doi:10.1038/ncomms10844)
Supplement: Supplementary Information — Supplementary Figures 1-6 and Supplementary Tables 1-3 [file ncomms10844-s1.pdf]

A

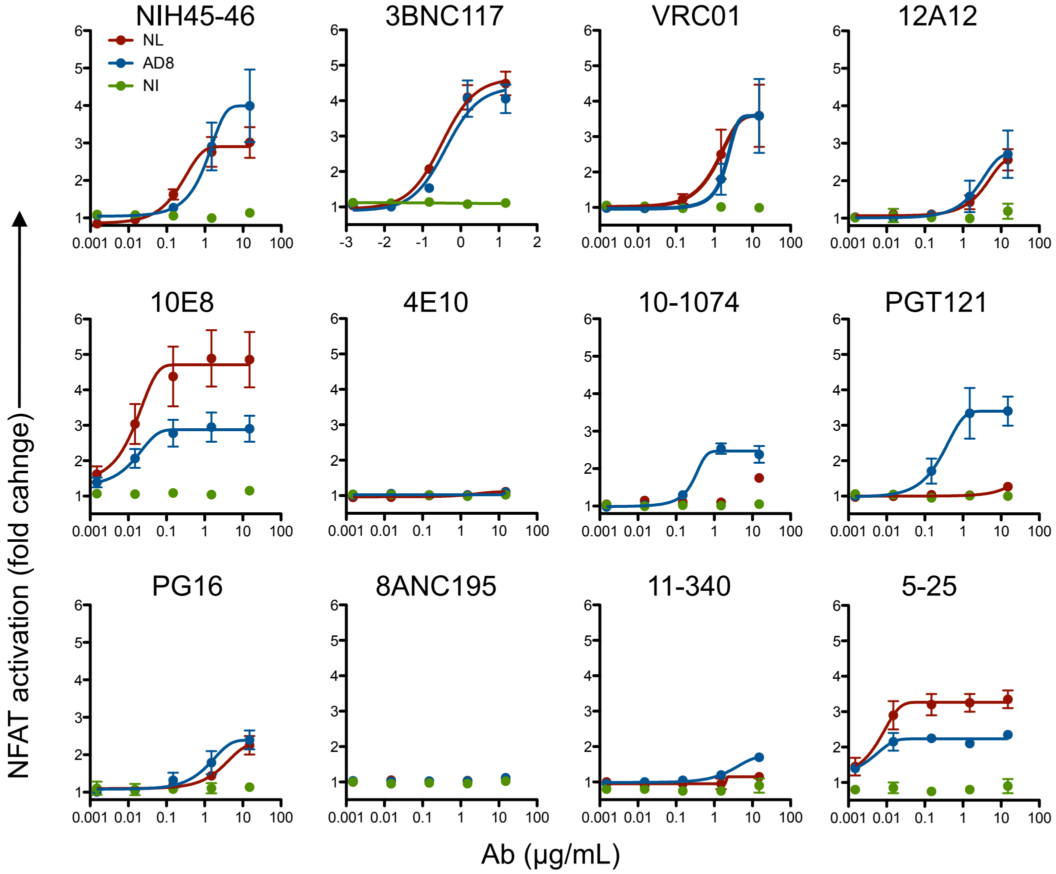

B

	EC50	
	NLAD8	NL4.3
3BNC117	0.3	0.2
NIH45-46	1,0	0.2
VRC01	4.2	1,0
12A12	2.2	3.1
PGT121	0.2	x
10-1074	0.3	x
10E8	<0.015	<0.015
4E10	x	x
PG16	0.2	x
8ANC195	x	x
5-25	<0.015	<0.015
11-340	x	x

C

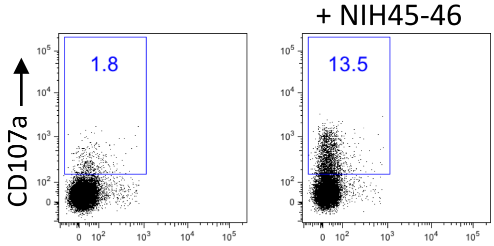

D

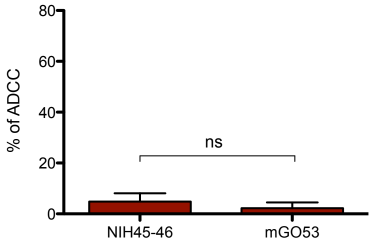

Supplementary Fig. 1. Identification of bNAbs that kill HIV-1 infected lymphocytes. (A) Analysis of the ability of bNAbs bound to infected cells to signal through FcγRIII. MT4C5 cells infected with HIV-1 (NLAD8 or NL4.3 strains) were incubated with the indicated antibodies and with a Jurkat indicator cell line expressing FcγRIII. Upon FcγRIII binding, activation of the NFAT transcription factor induces luciferase. After 18 hours of coculture, luciferase activity was measured. Top panel: Various antibody concentration (indicated in µg/mL) were tested. A mean of 3 independent experiments is shown. Error bars indicate SEM. NI: non-infected cells. **(B)** The corresponding EC50 were calculated using Prism software. (**C**) Analysis of NK cell degranulation in the ADCC assay. CEM-NKR cells infected with HIV-1 (NL4.3 strain) were incubated with NIH45-46 bNAb and with NK cells. After 6 hours, the % of NK cells expressing the CD107a degranulation marker (indicated in blue) was measured by flow cytometry. One representative experiment (out of 4) is shown. (**D**). Effect of NIH45-46 in the ADCC assay in the absence of NK cells. CEM-NKR cells infected with HIV-1 (NL4.3 strain) were incubated with NIH45-46 bNAb or with the mGO53 isotypic antibody. After 4 hours, the % of Gag+ CEM-NKR target cells was measured by flow cytometry. The mean of three independent experiments experiment is shown. Error bars indicate SEM (ns: non-significant, Wilcoxon test).

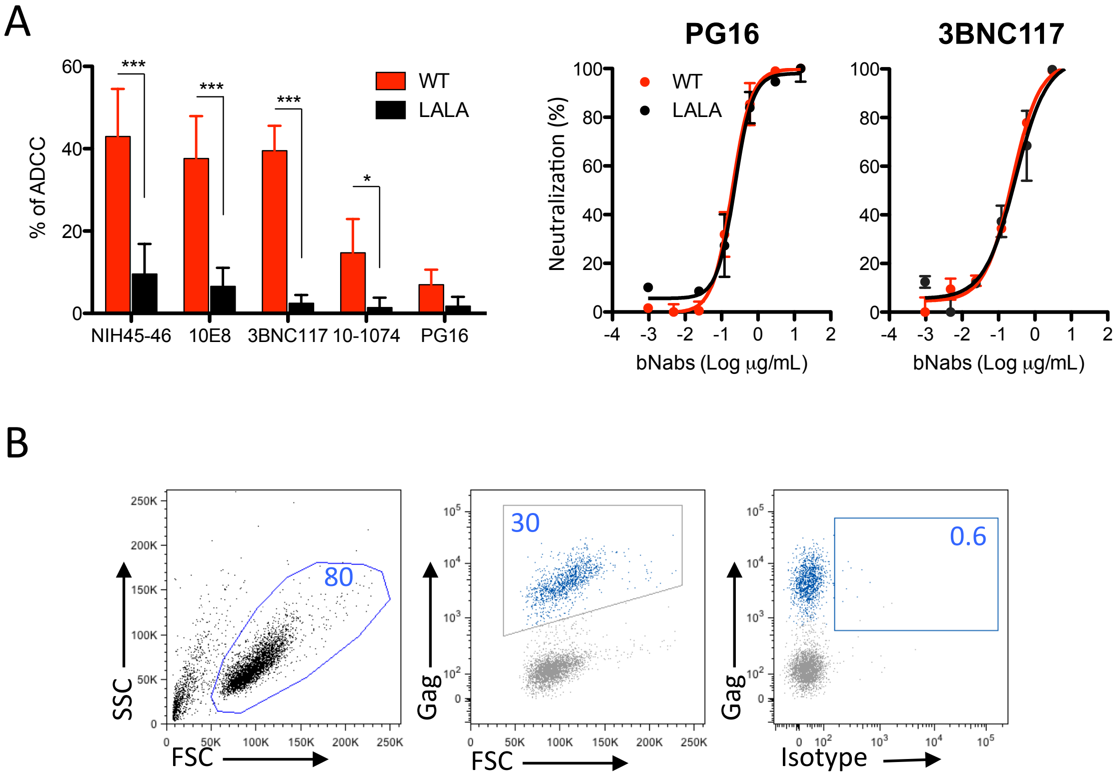

Supplementary Fig. 2. Characteristics of bNAbs (A) Analysis of ADCC competence and neutralizing activity of LALA bNAb mutants. The L234A-L235A (LALA) mutation, which abrogates Fc receptor binding, was introduced in the five indicated bNAbs. Left panel: CEM-NKR cells infected with HIV-1 (NL4.3 strain) were incubated with the indicated antibodies (1.5 µg/mL) and with NK cells. After 6 hours, the % of Gag+ CEM-NKR target cells was measured by flow cytometry. The % of ADCC was calculated as the disappearance of Gag+ cells (N= 3 experiments; error bars indicate SD and significance was determined by comparing each pair of wild-type and LALA antibody; ***, $p < 0.001$; **, $p < 0.01$; *, $p < 0.05$, Wilcoxon tests). The neutralizing activity of the antibodies, tested in the TzmBL assay, was not affected by the LALA mutations, as illustrated with PG16 and 3BNC117 (left panels, N= 3 experiments; error bars indicate SD of triplicates). **(B)** Gating strategy for the analysis of antibody binding to infected cells. CEM-NKR cells infected with HIV-1 were incubated with the indicated anti-Env or isotype human antibodies at 4°C and then fixed, permeabilized and stained for Gag expression. Cells were first gated on their FSC/SSC characteristics to select living cells (left panel). The Gag positive cells are highlighted in blue (middle panel). To define Env positivity, gates were set on the staining obtained with the mGO53 isotype control (right panel). The numbers indicate the % of positive cells in the depicted gates.

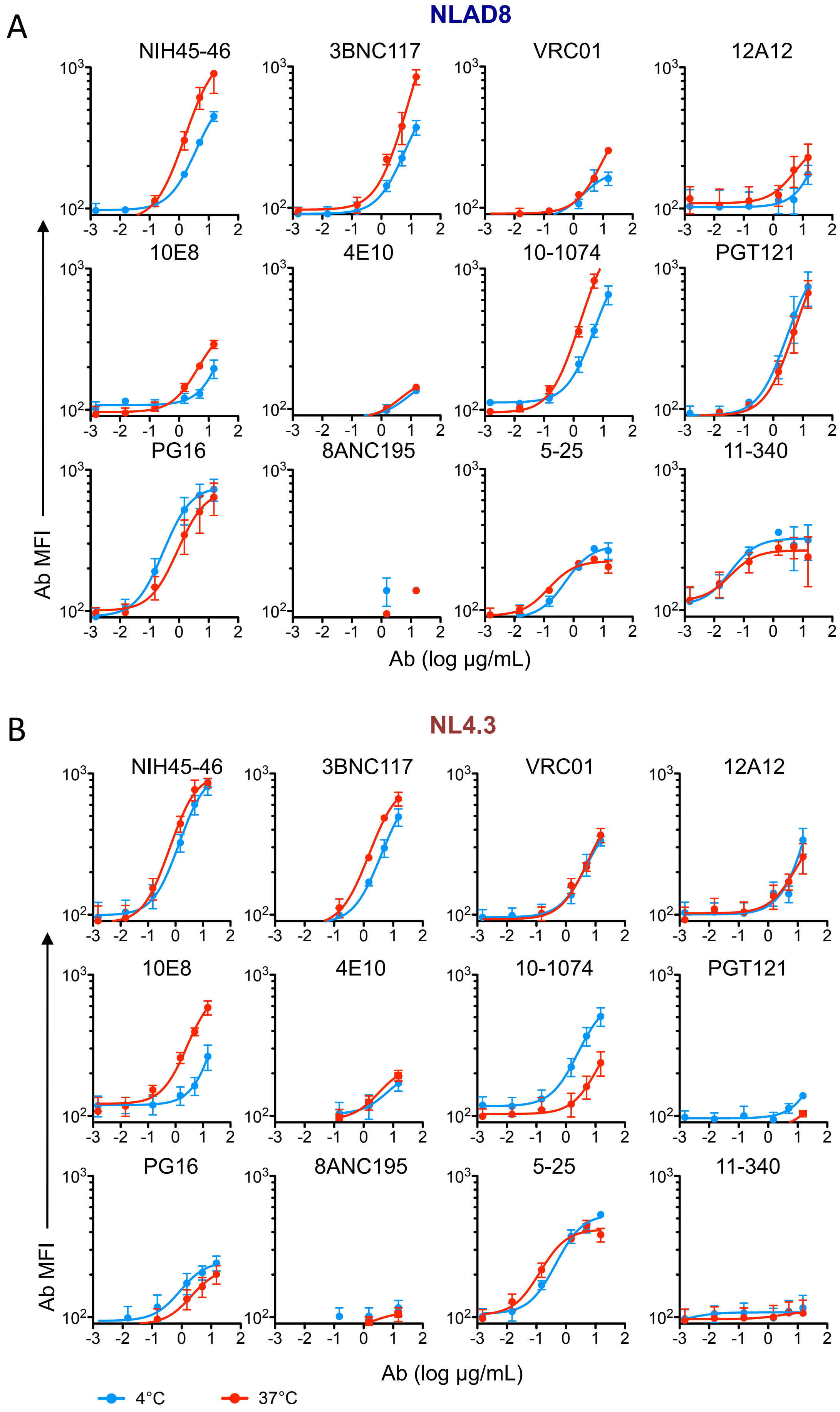
A
NLAD8
NIH45-46
3BNC117
VRC01
12A12
10E8
4E10
10-1074
PGT121
PG16
8ANC195
5-25
11-340
Ab MFI
Ab (log µg/mL)
B
NL4.3
NIH45-46
3BNC117
VRC01
12A12
10E8
4E10
10-1074
PGT121
PG16
8ANC195
5-25
11-340
Ab MFI
Ab (log µg/mL)
4°C
37°C

Supplementary Fig. 3. Dose response analysis of binding of bNAbs to HIV-1 infected lymphocytes. CEM-NKR cells infected with HIV-1 NLAD8 (A) or NL4.3 (B) were incubated with the indicated concentrations of antibodies at 4°C or 37°C and surface levels were analyzed by flow cytometry. The Median Fluorescence Intensity (MFI) of staining among Gag+ cells (Gag+) cells is shown (N= 3 independent experiments; Error bars indicate SEM).

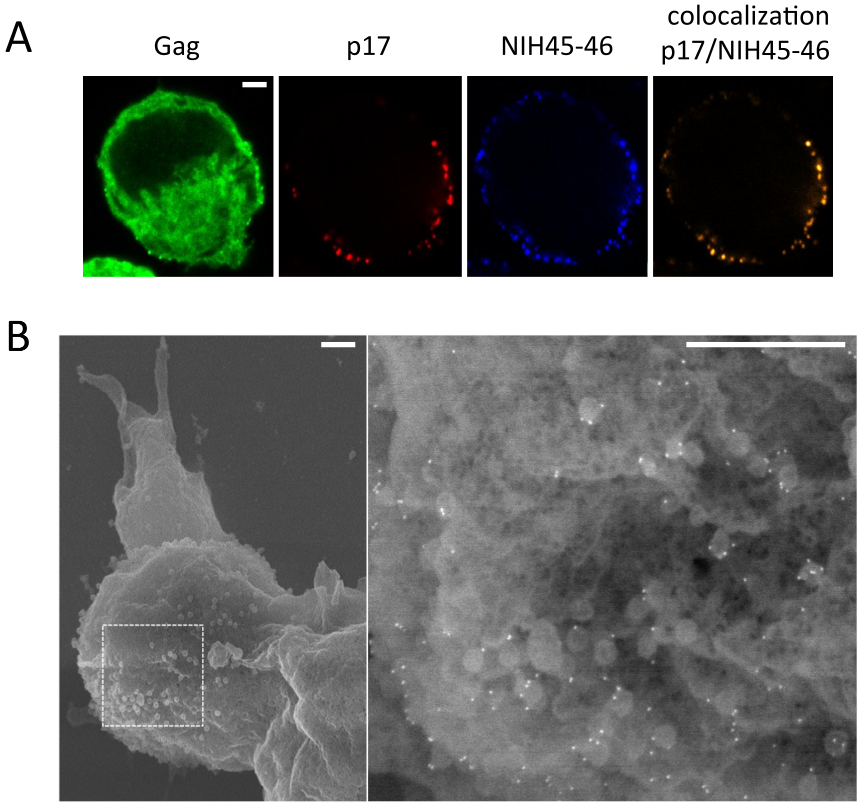

Supplementary Fig. 4. Binding of bNAbs at the surface of HIV-1 infected lymphocytes. (**A**) Localization of NIH45-46 bNAb and Gag at the cell surface analyzed by immunofluorescence. CEM-NKR cells infected with HIV-1 NL4.3 were stained with an anti-Gag p24 antibody (green, recognizing both Gag precursor and processed p24), with an anti-p17 (red, recognizing only processed p17 and thus staining viral budding regions at the cell surface) and with NIH45-46 (blue). One representative cell is shown. **(B)** Localization of NIH45-46 bNAb and Gag at the cell surface analyzed by scanning electron microscopy. CEM-NKR cells infected with HIV-1 (NLAD8 or NL4.3) were incubated with the indicated NIH45-46 bNAb and processed for immunogold and scanning electron microscopy. One representative cell is shown. Bar: 1 µm.

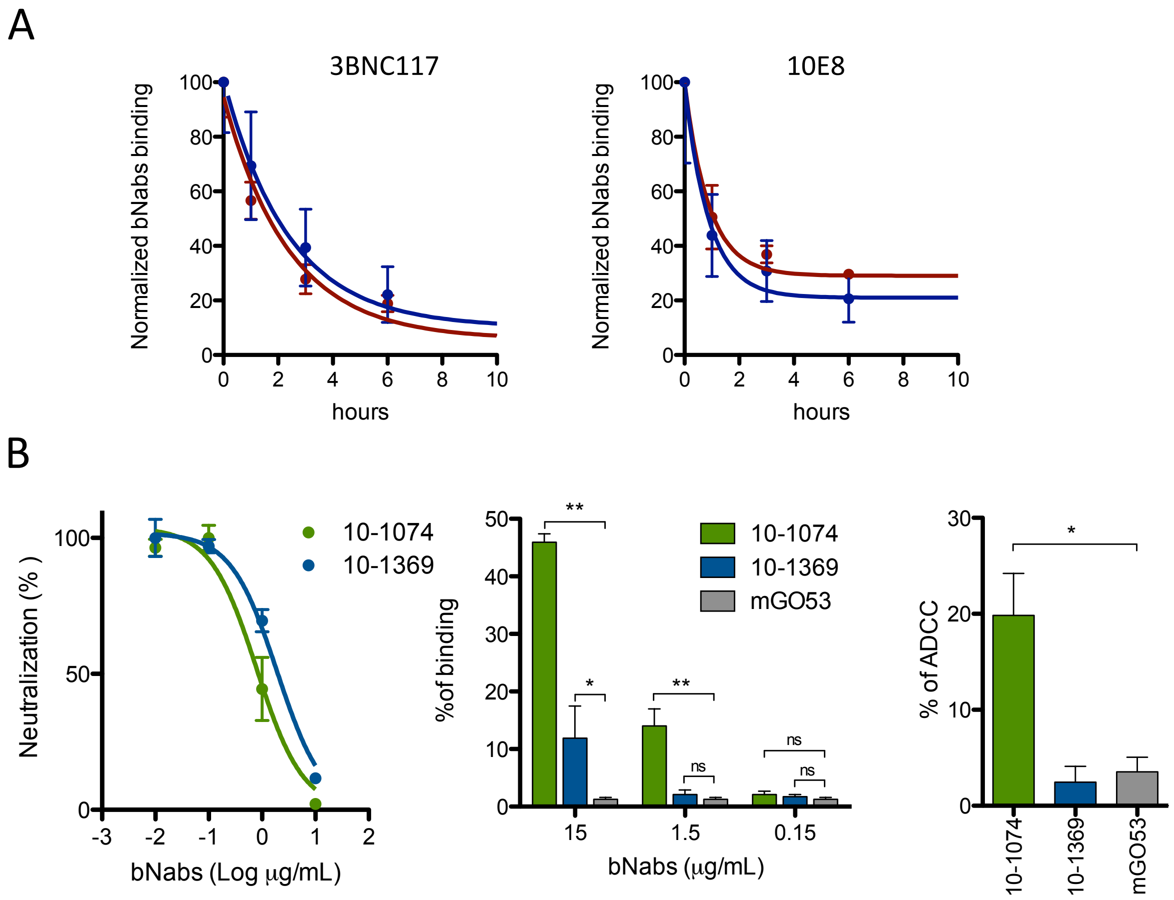

Supplementary Fig. 5. Binding, stability and ADCC activity of bNAbs with different affinities to HIV-1 Env. (A) Stability of 3BNC117 and 10E8 at the surface of infected cells. CEM-NKR cells infected with HIV-1 (NLAD8 or NL4.3) were incubated at 4°C with the indicated bNAbs and surface levels were analyzed by flow cytometry after the indicated incubation times at 37°C (N=3 experiments; error bars indicate SD and significance was determined by comparing NLAD8 and NL4.3 infected cells. For each antibody, there was no significant difference with the two viruses). (**B**) Analysis of the activity of 10-1074 and 10-1369, which targets the same epitope, but displays a 5-fold decrease of binding affinity to YU-2b gp140 trimers. Left panel: Neutralizing activity of the antibodies, tested in a TzmBL assay, against cell-free YU-2b virions. (N= 3 experiments; error bars indicate SD of triplicates). Middle panel: Binding of the antibodies to infected cells. CEM-NKR cells infected with HIV-1 (YU-2b strain) were incubated with the indicated doses of bNAbs and surface levels were analyzed by flow cytometry. The figures indicate the % of bNAb+ cells among infected (Gag+) cells. (N=3 experiments; error bars indicate SD). Right panel: ADCC activity of the antibodies. CEM-NKR cells infected with HIV-1 (YU-2b strain) were incubated with the indicated antibodies (1.5 µg/mL) and with NK cells. After 6 hours, the % of Gag+ CEM-NKR target cells was measured by flow cytometry. The % of ADCC was calculated as the disappearance of Gag+ cells (N= 3 experiments; error bars indicate SD, **, $p < 0.01$; *, $p < 0.05$, Wilcoxon test)

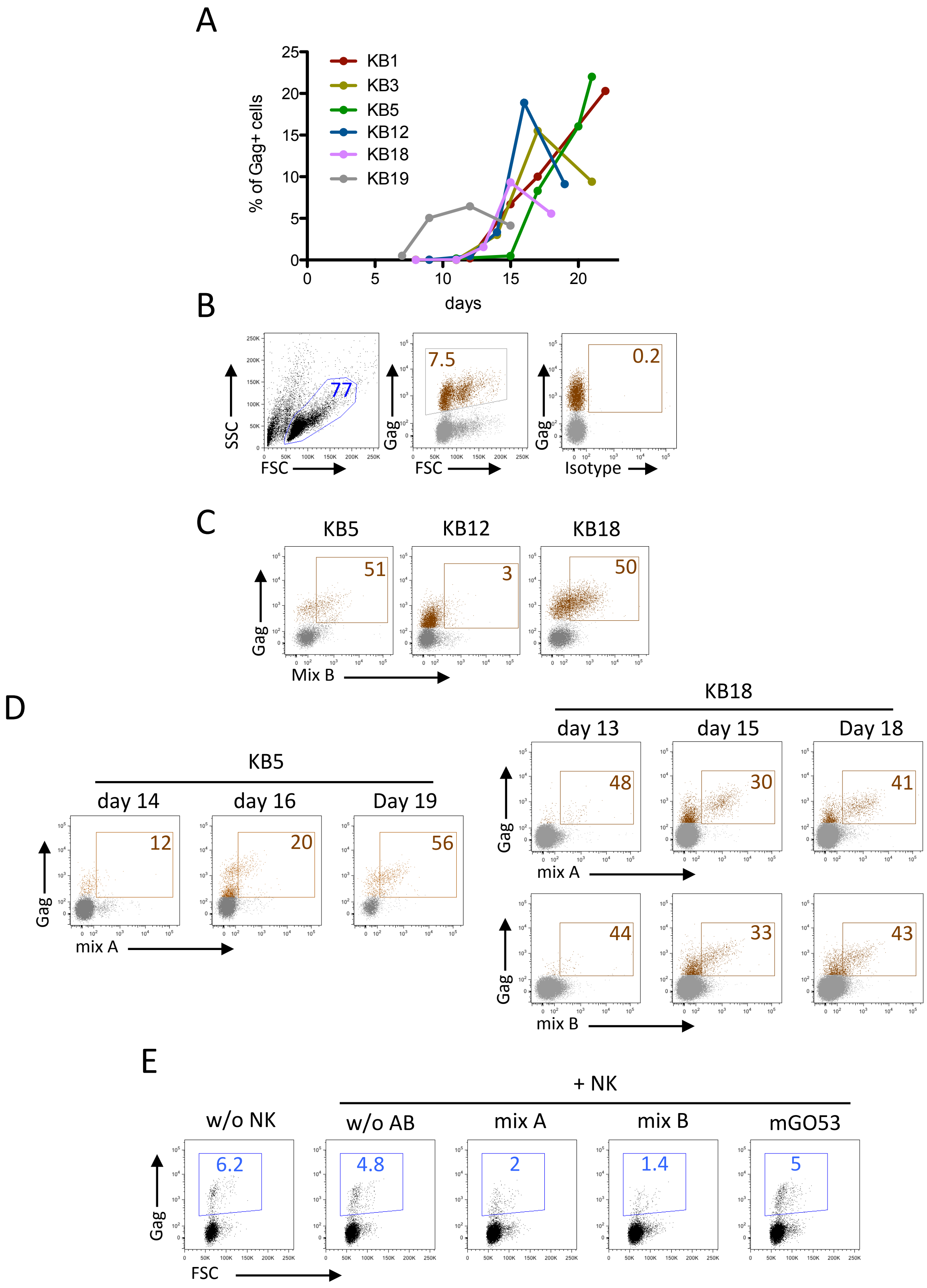
A
% of Gag+ cells
days
KB1
KB3
KB5
KB12
KB18
KB19
B
SSC
FSC
77
Gag
7.5
0.2
Isotype
C
KB5
KB12
KB18
51
3
50
Mix B
D
KB5
day 14
day 16
Day 19
12
20
56
mix A
KB18
day 13
day 15
Day 18
48
30
41
44
33
43
mix B
E
+ NK
w/o NK
w/o AB
mix A
mix B
mGO53
6.2
4.8
2
1.4
5

Supplementary Fig. 6. Binding and ADCC activity of bNAbs on reactivated HIV-1-infected cells from the viral reservoir. (A) HIV replication following in vitro reactivation. Purified CD4+ T cells were activated with PHA and IL-2 and viral replication was followed by flow cytometry at the indicated time points. The five positive samples are shown. Gag+ cells were not detected in the other patients. **(B)** Gating strategy for the analysis of antibody binding. Cells were first gated on their FSC/SSC characteristics to select living cells (left panel). The Gag positive cells are highlighted in brown (middle panel). To define Env positivity, gates were set on the staining obtained with the mGO53 isotype control (right panel). The numbers indicate the % of positive cells in the depicted gates. **(C)** Binding of the MixB combination of antibodies (VRC01; PGT121; 11-340 and 5-25) to cells from the three indicated patients. The numbers indicate the % of MixB+ cells among Gag+ cells. **(D)** Binding of MixA (NIH45-46; 3BNC117; 10E8; 10-1074 and PG16) or MixB combination of antibodies (VRC01; PGT121; 11-340 and 5-25) to cells from the three indicated patients, performed at the indicated times post-reactivation. The numbers indicate the % of MixA+ or MixB+ cells among Gag+ cells. **(E)** An example of ADCC activity of MixA and MixB against reactivated cells from patient KB18. Target cells were incubated with MixA or MixB and with NK cells. After 6 hours, the % of Gag+ target cells (blue numbers) was measured by flow cytometry. One representative experiment (out of 3 with this patient) is shown.

	AD8				NL			
	4°C		37°C		4°C		37°C	
	r=	p=	r=	p=	r=	p=	r=	p=
NIH45-46	0.94	0.02	0.94	0.02	0.99	0.003	0.99	0.003
3BNC117	1	0.003	0.94	0.02	0.94	0.02	0.94	0.02
10E8	0.83	0.06	0.94	0.02	0.83	0.06	1	0.003
PG16	0.8	0.1	0.8	0.13	-0.67	0.14	-0.7	0.14
5-25	0.89	0.03	0.89	0.03	0.83	0.06	0.94	0.02
10-1074	0.95	0.02	0.89	0.03	0.34	0.51	0.34	0.52
PGT121	0.9	0.03	0.83	0.06	-0.06	2	-0.6	0.24
VRC01	1	0.003	0.94	0.02	0.94	0.02	0.94	0.02
12A12	0.5	0.3	0.77	0.1	0.37	0.5	0.77	0.1
8ANC195	1	0.3	0.5	1	-1	0.33	-1	0.33
4E10	0.5	1	0.5	1	-0.5	1	-0.5	1
11-340	0.6	0.35	0.9	0.08	-0.44	0.42	-0.78	0.1

Supplementary Table 1. Correlation between ADCC competence of bNAbs and binding to infected cells. Dose-response analyses from Figure 4 were used to calculate the Spearman correlation coefficient between ADCC and binding at 4°C or 37°C. For each viral strain and the two temperatures of antibody binding on infected cells, Spearman's rho (r) and the p-value are shown. Significant p-values less than or equal to 0.05 are highlighted in red.

NLAD8

Ab	EC50 binding 4°C	EC50 binding 37°C	ADCC	Cell free neutralization
NIH45-46	2.2	0.9	0.5	0.3
3BNC117	4.3	2.3	1.3	0.15
10E8	/	2.6	3.6	1.8
PG16	0.23	0.6	0.07	0.12
5-25	0.2	0.3	0.6	X
10-1074	2.4	0.8	0.7	0.12
PGT121	5	2.3	0.4	0.21
VRC01	2.9	2.7	2.3	0.71
12A12	>15	3.1	>15	2.6
8ANC195	>15	>15	>15	5.7
4E10	>15	>15	>15	>15
11-340	0.007	0.006	0.01	14

NL4.3

Ab	EC50 binding 4°C	EC50 binding 37°C	ADCC	Cell free neutralization
NIH45-46	0.9	0.3	0.3	0.06
3BNC117	3,0	1.5	1.5	0.05
10E8	9.2	0.6	4.6	0.1
PG16	1	4.7	>15	0.7
5-25	0.3	0.2	0.2	X
10-1074	1.8	>15	>15	X
PGT121	>15	>15	>15	X
VRC01	5.4	3	2.1	0.2
12A12	10.6	10.6	6.8	9.4
8ANC195	>15	>15	>15	4
4E10	>15	>15	>15	4.3
11-340	>15	>15	>15	X

Supplementary Table 2. Efficacy of binding, ADCC and neutralization activity of the bNAbs. CEM-NKR cells infected with HIV-1 NLAD8 (upper table) or NL4.3 (lower table) were incubated with the indicated antibodies at 4°C or 37°C and surface levels were analyzed by flow cytometry. For each antibody, the efficacy of binding and of ADCC was calculated from the results presented in Figure 4. The neutralizing activity of the antibodies against cell-free HIV was tested in the TzmBL assay. Numbers indicate the EC50 (in µg/mL), defined as the effective concentration mediating 50% of the maximal effect. Data are mean of at least 3 independent experiments.

code	diagnosis (years)	ARV (years)	CD4 T Cell count	viremia	Viral Outgrowth Assay
KB1	30	11	457	<40	+
KB2	17	14	612	<40	-
KB3	26	8	345	<40	+
KB4	30	22	354	<40	-
KB5	28	22	252	<40	+
KB6	13	13	278	<40	-
KB7	25	7	451	<40	-
KB8	28	7	484	<40	-
KB9	30	7	454	<40	-
KB10	24	21	467	<40	-
KB11	4	4	370	<40	-
KB12	26	5	739	<40	+
KB13	26	23	412	<40	-
KB14	27	16	1049	<40	-
KB15	15	15	558	<40	-
KB16	15	11	356	<40	-
KB17	16	12	860	<40	-
KB18	24	9	2002	<40	+

Supplementary Table 3. Virological and biological characteristics of the 18 patients analyzed in this study. Cells from five patients (highlighted in yellow) were positive in the Viral Outgrowth Assay and were used for further analysis.
